# Local accessory gene sharing among Egyptian *Campylobacter* potentially promotes the spread of antimicrobial resistance

**DOI:** 10.1099/mgen.0.000834

**Published:** 2022-06-08

**Authors:** Shaimaa F. Mouftah, Ben Pascoe, Jessica K. Calland, Evangelos Mourkas, Naomi Tonkin, Charlotte Lefevre, Danielle Deuker, Sunny Smith, Harry Wickenden, Matthew D. Hitchings, Samuel K. Sheppard, Mohamed Elhadidy

**Affiliations:** ^1^​ Biomedical Sciences Program, University of Science and Technology, Zewail City of Science and Technology, Giza, Egypt; ^2^​ Milner Centre of Evolution, University of Bath, Claverton Down, Bath, UK; ^3^​ Chiang Mai University, Chiang Mai, Thailand; ^4^​ Swansea University Medical School, Swansea University, Swansea, UK; ^5^​ Department of Zoology, University of Oxford, Oxford, UK; ^6^​ Department of Bacteriology, Mycology and Immunology, Faculty of Veterinary Medicine, Mansoura University, Mansoura, Egypt; ^‡^​ Present address: Division of Virology, Department of Pathology, University of Cambridge, Tennis Court Road, Cambridge, UK; ^§^​ Present address: Nuffield Department of Medicine, Jenner Institute, University of Oxford, Oxford, UK

**Keywords:** accessory genome, antimicrobial resistance, *Campylobacter*, population structure

## Abstract

*
Campylobacter* is the most common cause of bacterial gastroenteritis worldwide, and diarrhoeal disease is a major cause of child morbidity, growth faltering and mortality in low- and middle-income countries. Despite evidence of high incidence and differences in disease epidemiology, there is limited genomic data from studies in developing countries. In this study, we aimed to quantify the extent of gene sharing in local and global populations. We characterized the genetic diversity and accessory-genome content of a collection of *Campylobacter* isolates from the Cairo metropolitan area, Egypt. In total, 112 *Campylobacter* isolates were collected from broiler carcasses (*n*=31), milk and dairy products (*n*=24), and patients suffering from gastroenteritis (*n*=57). Among the most common sequence types (STs), we identified the globally disseminated host generalist ST-21 clonal complex (CC21) and the poultry specialists CC206, CC464 and CC48. Notably, CC45 and the cattle-specialist CC42 were under-represented, with a total absence of CC61. Core- and accessory-genome sharing was compared among isolates from Egypt and a comparable collection from the UK (Oxford). Lineage-specific accessory-genome sharing was significantly higher among isolates from the same country, particularly CC21, which demonstrated greater local geographical clustering. In contrast, no geographical clustering was noted in either the core or accessory genome of CC828, suggesting a highly admixed population. A greater proportion of *Campylobacter coli* isolates were multidrug resistant compared to *Campylobacter jejuni *. Our results suggest that there is more horizontal transfer of accessory genes between strains in Egypt. This has strong implications for controlling the spread of antimicrobial resistance among this important pathogen.

## Data Summary

Short-read data are available from the National Center for Biotechnology Information (NCBI) Sequence Read Archive, associated with BioProject PRJNA576513 (https://www.ncbi.nlm.nih.gov/bioproject/PRJNA576513). Assembled genomes and supplementary material are available from FigShare (https://doi.org/10.6084/m9.figshare.19927469 [1]); individual accession numbers and assembled genome statistics are given in Table S1 (available with the online version of this article). Phylogenetic trees can be visualized and manipulated on Microreact for the whole dataset: https://microreact.org/project/Campy-Egypt.

**Impact Statement**

In this paper, we describe genomic evidence for the spread of antimicrobial resistance among *Campylobacter* isolates from Egypt. We found that more *Campylobacter coli* isolates were multidrug resistant compared to *Campylobacter jejuni* and that horizontal transfer of accessory genes was spreading resistance genes between strains in Egypt. This suggests that differences in food production systems and antimicrobial usage may impact on the emergence of resistance in important pathogens.

## Introduction

Diarrhoeal disease is a major cause of child morbidity, growth faltering and mortality in low- and middle-income countries (LMICs) [2, 3]. *Campylobacter* is the most common cause of bacterial gastroenteritis worldwide [4] and typically human campylobacteriosis is readily diagnosed as a disease associated with consumption of contaminated food, especially poultry [5, 6]. Extremely high incidence in LMICs, high exposure rates [7] and endemism among young children suggests a different epidemiology [4, 8, 9]. Frequent or chronic (re)infection is allied to significant morbidity, cognitive development impairment and even death [10–13]. In Egypt, campylobacteriosis is common and a leading cause of paediatric diarrhoea, with an incidence of 1.2 episodes per child, per year [14, 15] and up to 85% of children infected in their first year [16]. Despite the high frequency of reported cases of *Campylobacter *-associated diarrhoea in Egypt [14], there are no detailed surveillance studies on the dominant sequence types (STs) and proliferation of genotypes associated with the onset of post-infectious sequelae, such as irritable bowel syndrome, Guillain-Barré syndrome or Miller Fisher syndrome [17].

*Campylobacter* species are often part of the gut microbiota of various wild and farmed animals, leading to frequent contamination of human food products [18, 19]. In Egypt, farming practices often lack adequate biosecurity and regulation. Only limited studies have reported the prevalence and distribution of *Campylobacter* in Egyptian campylobacteriosis cases [4], and little is known about the dominant source reservoirs driving infection and transmission [20]. In Europe, potential source reservoirs have been identified through source attribution studies, with poultry products regarded as the primary source of infection [6, 21–23]. Host-adaptation of *Campylobacter* to a wide range of hosts is reflected in its population structure [24–28], with many common human infection lineages able to infect multiple host species. These host generalist lineages include *Campylobacter jejuni* ST-21, ST-45 clonal complexes (CCs) and the *Campylobacter coli* ST-828 complex [22, 25]. Other genotypes are only found in a single reservoir species, often associated with global poultry or cattle production. Host specialist CCs common in human disease include the poultry-associated ST-353, ST-354 and ST-257 [6, 29], and cattle specialist ST-61 [30, 31].

Human infection in developed countries is typically sporadic and self-limiting, not requiring treatment with antibiotics. However global rates of antimicrobial resistance (AMR) are rising [31, 32] in line with other Gram-negative gastrointestinal pathogens [33, 34]. Widespread agricultural usage has also driven the proliferation of tetracycline resistance through its use as a growth promoter in some food processing animals [35, 36]. In particular, *C. coli* has shown an ability to acquire erythromycin-resistance genes from other species [31]. This has not been explored for Egyptian *Campylobacter* isolates, where agricultural antibiotic usage is poorly regulated [37] and self-medication for gastrointestinal disease is common [38, 39]. Global differences in the use of quinolones are also likely responsible for the geographical differences observed in quinolone resistance [40–42].

Here, we aim to better understand *Campylobacter* epidemiology in countries with differences in farm animal husbandry, slaughter, meat sale and consumption. Based on our recent study reporting the AMR phenotypes in this population [43], we aimed to quantify accessory-genome sharing among divergent lineages in well sampled *Campylobacter* populations from Egypt and the UK. By screening genome content, including virulence and AMR determinants, we show enhanced local gene sharing between divergent lineages is greater in Egypt, which may be linked to the spread of AMR. This provides a basis for considering complex transmission networks in LMICs and highlights the importance of globally transmitted AMR *Campylobacter* lineages.

## Methods

### Isolate collection

In total, 112 *Campylobacter* isolates were collected in Cairo, Egypt, from September 2017 to December 2018, including 31 isolates from broiler carcasses, 24 isolates from milk and dairy products, and 57 clinical isolates. The clinical isolates were obtained from stool samples of patients admitted to two different hospitals in Northern Giza (Egypt) suffering from gastroenteritis symptoms. A total of 31 isolates were recovered from 87 samples obtained from Imbaba General Hospital and 26 recovered from 59 samples obtained from Al Haram Hospital. Patient names were anonymous. Among 146 stool specimens collected, the prevalence of *Campylobacter* spp. among patients suffering from gastroenteritis was 39 % (57/146) as confirmed at the genus level by culture, biochemical and microscopic examination, and PCR amplification of the 16S rRNA gene [44]. *C. jejuni and C. coli* were differentiated at the species level by PCR of the *mapA* gene [45] and *ceuE* gene [46], respectively. A questionnaire was distributed to all admitted patients requesting details of clinical presentation (e.g. duration of illness, symptoms, medication prescribed), dietary record of the previous 2 weeks, including consumption of specific or undercooked meats or unpasteurized milk, exposure to animal manure or faeces, and any retail outlets commonly used by patients for food consumption prior to the onset of illness. Based on this questionnaire, food samples were obtained from different small retail outlets in close proximity with the study hospitals (19 stores for broiler carcasses and 25 stores for dairy products), following a stratified randomized sampling strategy. Briefly, each district surrounding the two hospitals served as a stratum and, within each district, simple random sampling from the stores was conducted. Store names were de-identified, and no permits were required for food sample collection. All samples were transferred within 2–4 h in an ice box for microbiological analysis. The prevalence rates of *Campylobacter* species among samples broiler carcasses and dairy products were 27.7% (31/112) and 13.8% (24/174), respectively.

### Sample culturing and whole-genome sequencing

The isolation and enumeration of *Campylobacter* strains from different food matrices intended for human consumption was performed according to the ISO 10272–1 (selective enrichment method) with minor modifications. In brief, samples were seeded into 90 ml Bolton selective enrichment broth (Oxoid) and incubated at 37 °C for 4 h, followed by further incubation at 42±1 °C for 48 h under microaerophilic conditions. The enrichment broth cultures were plated onto modified charcoal cefoperazone deoxycholate agar (m-CCDA agar; Oxoid). Plates were incubated at 42±1 °C in microaerophilic conditions using AnaeroGen 2.5L sachets (Oxoid) for 40 to 48 h. Following incubation, viable *Campylobacter* colonies were counted using serial dilutions. Subculturing of colonies presumed to be *Campylobacter* was done on Columbia blood agar (Oxoid), and confirmation was performed using morphological characters, oxidase test and PCR amplification of the 16S rRNA genes (for molecular identification at the genus level), and *mapA* and *ceuE* genes (for molecular differentiation of *C. jejuni and C. coli* at the species level, respectively). For *Campylobacter* isolation from human patients, stool samples were cultured on m-CCDA agar. Plates were incubated at 42±1 °C under microaerophilic conditions usingAnaeroGen2.5L sachets for 48 h. All isolates were subcultured from −80 °C frozen stocks onto Mueller-Hinton agar (Oxoid). Plates were incubated at 42±1 °C under microaerophilic conditions using AnaeroGen 2.5L sachets. Following enumeration step, we picked one colony from each plate for subculture and confirmation. It is possible that more than one species, CC or ST is present in the same sample and methods exist for investigating multi-lineage samples [47]. However, here, we use the more common single colony approach that assumes that the first single colony that is picked likely represents one of the most common lineages in that sample.

As previously described [43], genomic DNA was extracted from 112 Egyptian isolates using the QIAamp DNA mini kit (Qiagen), according to the manufacturer’s instructions, and concentrations were quantified using a NanoDrop spectrophotometer before genome sequencing using an Illumina MiSeq. Nextera XT libraries (Illumina) were prepared following the manufacturer’s protocols and short paired-end reads were sequenced using a 2×300 bp paired end v3 reagent kit (Illumina).

### Genome datasets

**Fig. 1.**
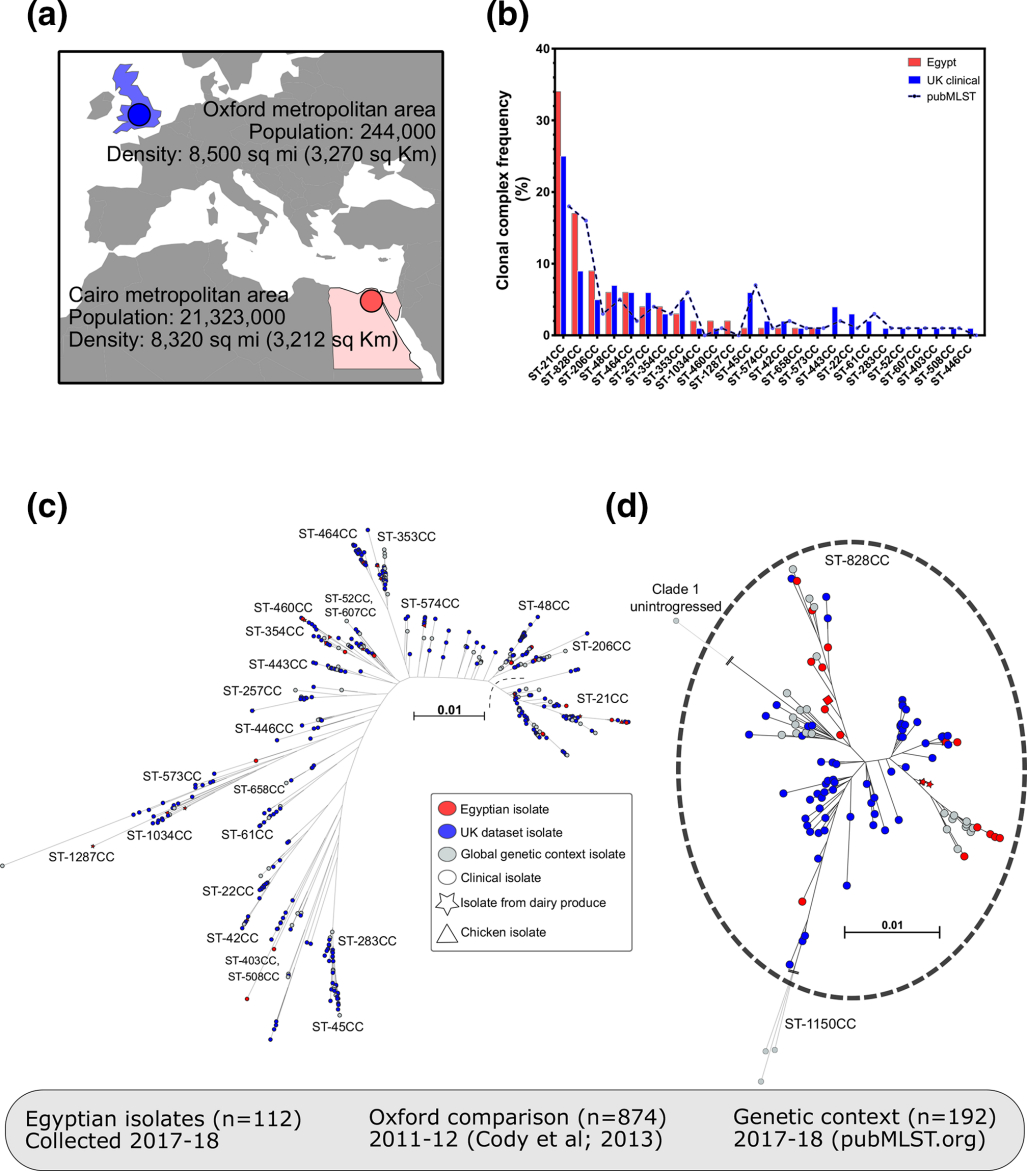
(**a**) Demographic data for Cairo, Egypt, from which we collected *Campylobacter* spp. isolates (*n*=112; red circle) from clinical cases, broiler carcasses and dairy products collected over a 14 month sampling period. Our collection was compared to a similar published survey from Oxford, UK (*n*=874; blue circle) [53, 54]; and isolates from pubMLST.org (*n*=192) for additional genetic context. (**b**) CCs of isolates collected from Cairo were ranked according to the frequency in our local dataset and how often they have been sampled from human disease isolates (data from pubMLST; https://pubmlst.org/). Alignments were made from concatenated gene sequences of all core genes (found in ≥95 % isolates) using mafft version 7 [59], on a gene-by-gene basis. Separate maximum-likelihood phylogenies were reconstructed with a GTR+I+G substitution model and ultra-fast bootstrapping (1000 bootstraps) [60] implemented in iq-tree (version 1.6.8) [61] for (**c**) *C. jejuni* (*n*=1048) and (**d**) *C. coli* (*n*=132), and visualized on Microreact (https://microreact.org/project/Cjejuni_Egypt; https://microreact.org/project/Ccoli_Egypt) [62]. Scale bars represent genetic distance of 0.01.

Raw sequencing reads were quality trimmed and filtered using Trimmomatic (version 0.33) [48], and genomes assembled *de novo* using SPAdes (version 3.8.0) [49]. The mean number of contigs was 72 (range: 12–471) for a mean total assembled sequence size of 1.70 Mbp (range: 1.56–1.86 Mbp). The mean N50 contig length (L50) was 14577 (range: 3794–55 912) and the mean G+C content was 30.8 % (range: 30.5–31.6 %). We augmented our collection by assembling a context dataset of previously published isolates (*n*=204) to represent the known diversity of *C. jejuni* and *C. coli* [28, 50–52]. In addition, we also compared our single city survey with a previously published survey of clinical isolates from Oxford in the UK (*n*=874 isolates collected over 1 year) [53]. The Oxford data was selected to be matched, as much as possible, with the Cairo dataset in terms of population density (Fig. 1). Of course, this is not perfect. However, well characterized *Campylobacter* isolates collected in Oxford from 2011 to 2012 (https://pubmlst.org/organisms/campylobacter-jejunicoli), a subset of which have been described by Cody *et al*. [54], offered the opportunity to compare genomes from different populations. Simpson’s index showed that the datasets had similar diversity.

### Core-genome characterization

Isolate genomes were archived in BIGSdb and multilocus sequence typing (MLST) STs derived through blast comparison with the pubMLST database [52, 55–58]. An alignment of all 112 *Campylobacter* isolates was constructed from concatenated gene sequences of all core genes (found in ≥95 % isolates) using mafft (version 7) [59], on a gene-by-gene basis. A maximum-likelihood phylogeny was reconstructed for *C. jejuni* and *C. coli* isolates combined using a GTR+I+G substitution model and ultra-fast bootstrapping (1000 bootstraps) [60] implemented in iq-tree (version 1.6.8) [61], and visualized on Microreact (https://microreact.org/project/Campy-Egypt) [62]. Simpson’s index of ST diversity was calculated for the Cairo and Oxford datasets using the equation:

$$D=1-\frac{\sum n\left(n-1\right)}{N\left(N-1\right)}$$

where *n* is the number of isolates of each ST and *N* is the total number of isolates [63].

### Quantifying accessory-genome variation

All unique genes present in at least one isolate (the pangenome) were identified by automated annotation using prokka (version 1.13) [64]; followed by pirate, a pangenomics tool that allows for orthologue gene clustering in bacteria [65]. We defined genes in pirate using a wide range of amino acid percentage sequence identity thresholds for Markov cluster algorithm (MCL) clustering (45, 50, 60, 70, 80, 90, 95, 98). Genes in the pangenome were ordered initially using the NCTC 11168 reference, followed by the order defined in pirate based on gene synteny and frequency [66, 67]. As described previously, a matrix was produced summarizing the presence/absence and allelic diversity of every gene in the pangenome list, with core genes defined as present in 95 % of the genomes and accessory genes as present in at least one isolate . This pangenome matrix was visualised using phandango, alongside a core genome phylogeny (Fig. S1) [68, 69]. Pairwise core- and accessory-genome distances were compared using PopPUNK (version 2.1.1) [70], which uses pairwise nucleotide *k*-mer comparisons to distinguish shared sequence and gene content to identify divergence of the accessory genome in relation to the core genome. A two-component Gaussian mixture model was used to construct a network to define clusters, comparable to other *Campylobacter* studies [71].

Core-genome variation between isolates was quantified by calculating the pairwise average nucleotide identity (ANI) of all (*n*=112+874) *Campylobacter* genomes using FastANI v.1.058 [72]. The gene presence matrix produced by pirate was used to generate a heatmap of shared pairwise accessory-genome genes. Averages were calculated for within and between country comparisons in addition to focussed analysis on the ST-21 (* C. jejuni *) and ST-828 (* C. coli *) CCs. Antimicrobial-resistance genes and putative virulence genes were detected through comparison with reference nucleotide sequences using ABRicate (version 0.8) (https://github.com/tseemann/abricate) and the VFDB (Virulence Factor Database) and NCBI (National Center for Biotechnology Information) database, respectively [73, 74]. Point mutations related to antibiotic-resistance genes were identified by PointFinder [75] using the star-amr software package (https://github.com/phac-nml/staramr).

## Results

### Globally circulating genotypes among Egyptian *Campylobacter* isolates

We sequenced and characterized a collection of *Campylobacter* spp. isolates (*n*=112) from clinical cases, broiler carcasses and dairy products collected over a 14 month sampling period in Cairo, Egypt (Fig. 1a, Table S1). Isolate genotypes were compared with all genomes deposited in the pubMLST database (97 012 profiles, data accessed 17th February 2020) and ranked according to how frequently they were found associated with human disease (Fig. 1b). Egyptian *C. jejuni* isolates belonged to 15 CCs with diverse assemblage of STs. Nearly half of the isolates (*n*=29, 47%) were from common lineages, isolated many times before and recorded in pubMLST (>50 MLST profiles; Fig. 1b), with the globally disseminated lineages of ST-21 CC (*n*=37, 41%), ST-206 CC (*n*=10, 11%) and ST-464 CC (*n*=7, 8%) the most abundant. Several other poultry-associated CCs, which are common in human disease [29, 76], including ST-353 (*n*=3, 3.2%,), ST-354 (*n*=4, 4.3%) and ST-257 (*n*=4, 4.3%), were identified. Other globally disseminated lineages were found less often in Egypt (*n≤*2), i.e. ST‐460 (*n*=2), ST‐1034 (*n*=2), ST‐42 (*n*=1), ST-45 CC (*n*=1), ST‐573 (*n*=1), ST‐574 (*n*=1) and ST‐658 (*n*=1) [77, 78].

### Local STs

Comparison with a collection representing the known genetic diversity of *C. jejuni* and *C. coli* identified some common STs (>1000 profiles in pubMLST) that were completely absent in our Egyptian collection, i.e. ST-53 and ST-829 (* C. coli *), and ST-22, ST-61, ST-51 and ST-1068 (* C. jejuni *) (Fig. 1c, d). Two isolates belonging to ST-1287 CC, a genotype that has previously been isolated from poultry and the environment [79], were observed exclusively among our Egyptian isolates, yet absent in UK and global datasets. Furthermore, there were also some STs belonging to ST-21 CC that were found in the Egyptian isolate collection (*n≥*3) that are rare in global collections (<100 profiles in pubMLST), i.e. ST-1519 (*n*=4) and ST-3769 (*n*=3). It was also observed that more *C. coli* were found among Egyptian clinical isolates than is typically observed, specifically the *C. coli* lineage ST-828 CC (Table S1), 90.4% *C *. *coli* isolates (19/21) belonged to the ST-828 CC within the Egyptian dataset, and two *C. coli* isolates with unassigned CC of STs, ST-7951 and ST-1681. Three rare STs belonging to ST-828 CC were exclusively found in the Egypt dataset, which are ST-1058 (*n*=1), ST-1059 (*n*=1) and ST-7950 (*n*=1) (Table S1).

### Increased sharing of accessory genes contributes to a local gene pool

Our Egyptian dataset was compared directly with a previously published study of a single city, ~1 year survey from Oxford in the UK. Both populations were similarly diverse, specifically there were 50 STs (16 CCs) among the Egyptian isolate collection, with a Simpson’s diversity index of 0.817, compared to 205 STs (32 CCs) among the Oxford collection of genomes (Simpson’s diversity index=0.895; Fig. 1c, d). Therefore, as phylogeographic population structuring is known not to be a major factor in *Campylobacter* (compared to host) [41, 71, 80], major differences identified using Simpson’s index are likely linked to other factors such as different food production systems.

**Fig. 2.**
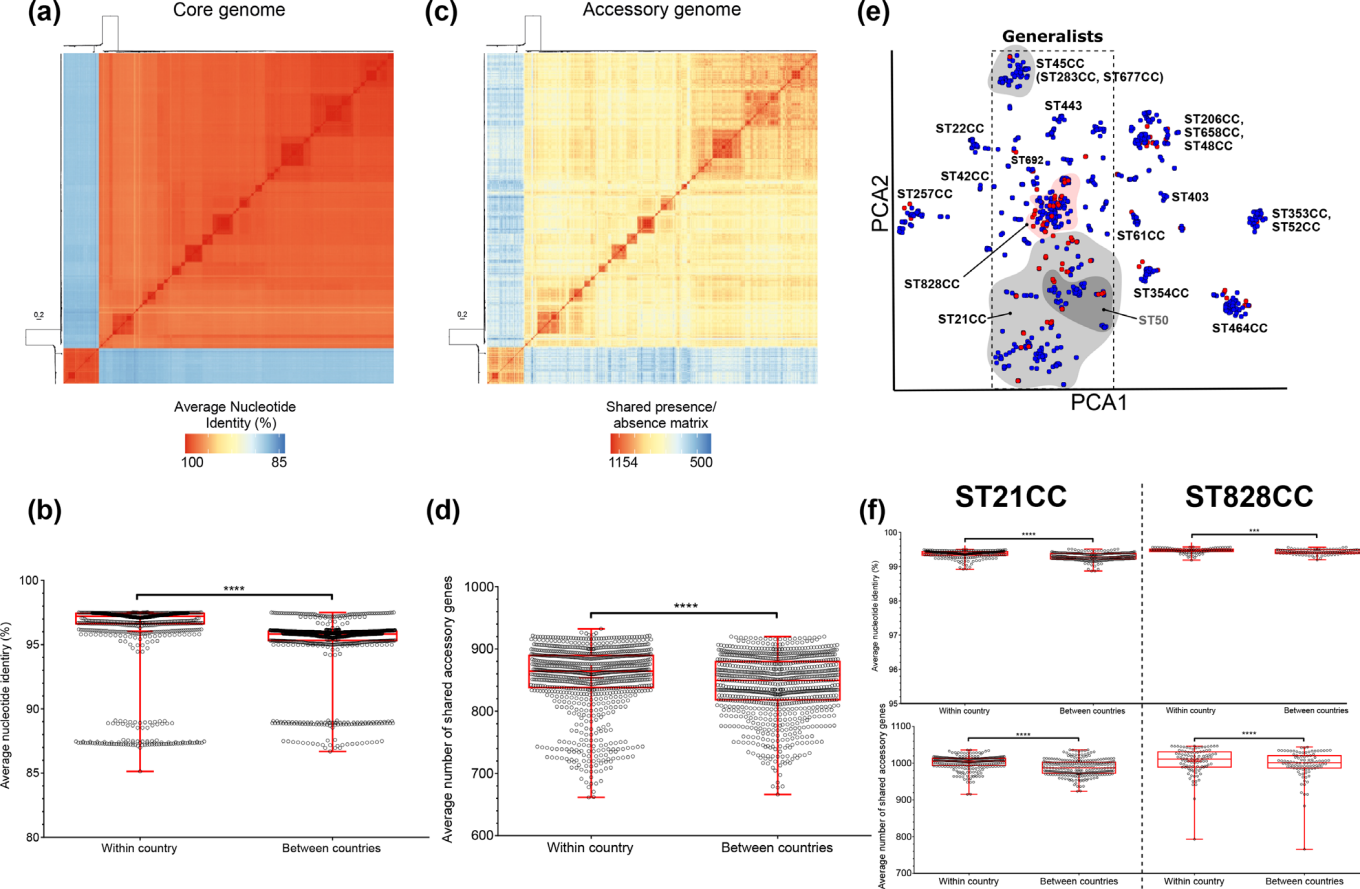
(**a**) Core-genome variation between isolates was quantified by calculating the pairwise ANI of all UK and Oxford *Campylobacter* genomes (*n*=112+874) using FastANI v.1.058 [72]. (**b**) The ANI for each isolate was estimated and averages compared within and between countries. (**c**) The gene presence matrix produced by pirate was used to generate a heatmap of shared pairwise accessory-genome genes. (**d**) Averages were calculated for within and between country. (**e**) Clustering of pairwise core- and accessory-genome distances were compared using PopPUNK. Interactive visualization on Microreact: https://microreact.org/project/Campy-Egypt. (**f**) Comparisons of within and between country ANI and accessory-gene sharing were also analysed for our two most common Egyptian lineages, ST-21 (* C. jejuni *) and ST-828 (* C. coli *) CCs.

First, we compared population-wide accessory-genome diversity using pirate to construct a pangenome of all Egyptian and Oxford isolates (*n*=986). Consistent with other studies, we identified an open pangenome, meaning that the number of genes in the pangenome continues to increase with each additionally sequenced isolate. Accessory genes represented nearly three-quarters of the pangenome (3781 genes; 74 % of pangenome) with a quarter of the genes identified (1336, 26%) considered core genes present in 95 % or more of the isolates (Fig. S1, Table S2). Pairwise comparison of the core nucleotide sequence (%ANI) and accessory-genome sharing of all isolates reflected the clonal frame, with clusters of closely related isolates sharing a large percentage of ANI, seen as darker blocks on the diagonal of the heat maps. This indicates high sequence similarity among isolates of the same CC (Fig. 2a, b). Direct comparison between the Oxford and Cairo datasets suggested an increase in within-country, local accessory gene sharing (Fig. 2c, d). The structured clustering of pairwise comparisons of shared accessory genes suggested that this may vary between lineages and visualization of the differences in the distribution of pairwise genomic distances with PopPUNK also pointed towards lineage-specific shared gene pools (Fig. 2e). Box plots (Fig. 2b, d) show that the mean similarity between core and accessory genome is significantly greater within country. Repeating these analyses for the two most common CCs, ST-21 CC and ST-828 CC, again showed greater genome similarity within country (Fig. 2f).

### Locally diverged STs within the globally disseminated ST-21 CC

**Fig. 3.**
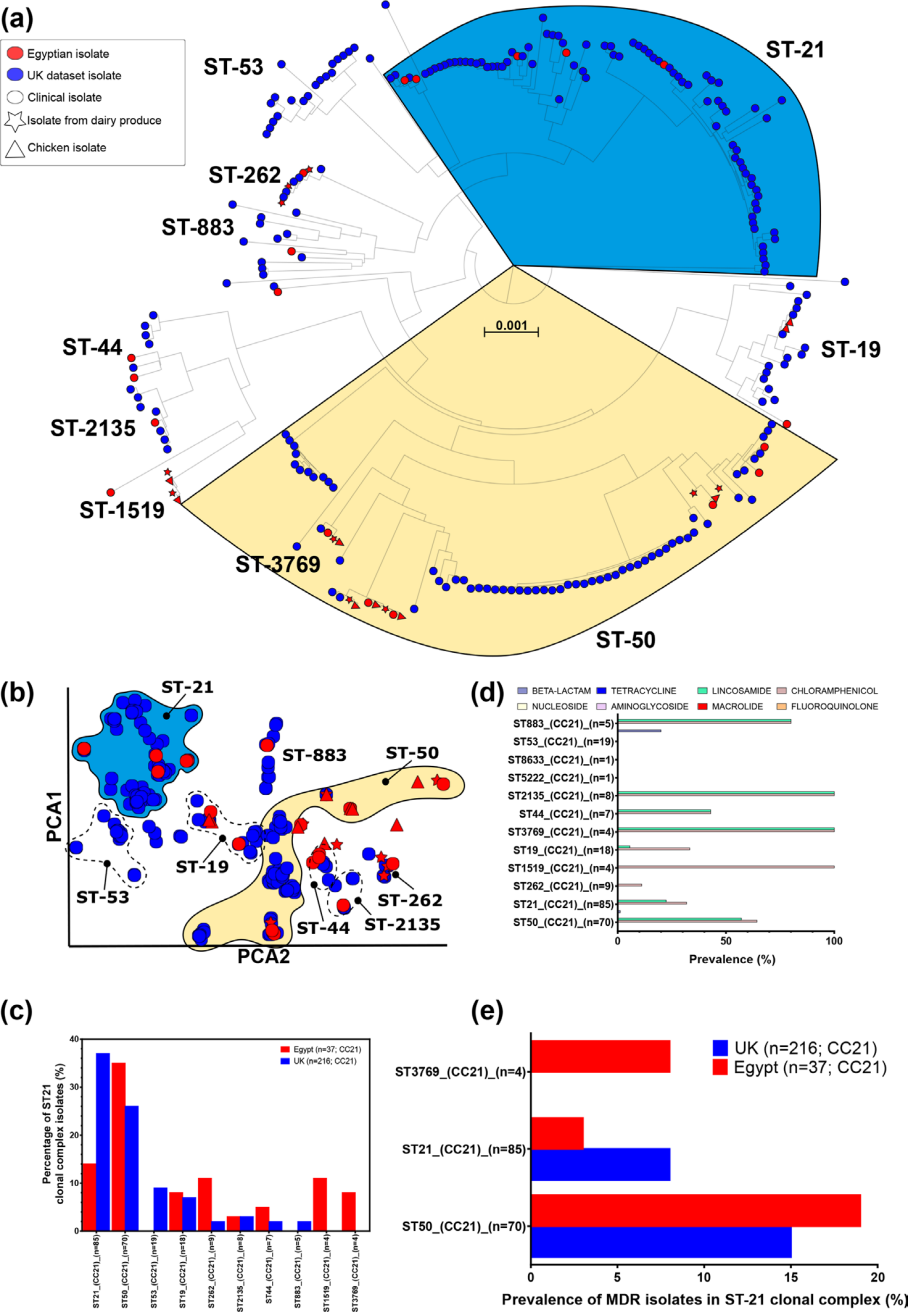
(**a**) Sub-tree of all Egyptian and UK ST-21 CC (CC21) isolates (*n*=251). Common STs are annotated and ST-50 (yellow) and ST-21 (blue) are highlighted. Scale bar indicated genetic distance. (**b**) Within CC clustering of pairwise core- and accessory-genome distances with PopPUNK according to two priciple components (PCA). (**c**) Prevalence of the most common STs found within CC21. (**d**) Prevalence of AMR determinants grouped by antibiotic class for each CC21 ST. (**e**) Prevalence of MDR isolates (AMR determinants for three or more antibiotic classes) in CC21 STs.

As one might expect of within lineage (CC) comparisons, all ST-21 CC isolates shared more than 99 % core-genome nucleotide identity and shared more accessory genes than the population average (852 genes; Fig. 2c, f) and significantly more genes were shared between isolates from the same country (*t*-test with Welch correction; *P*<0.0001). A maximum-likelihood phylogeny of all CC21 isolates (*n*=251), the most common CC identified in our collection from Cairo, identified geography-specific clusters of isolates (Fig. 3a). These clones also clustered together when visualizing the distribution of pairwise genomic distances with PopPUNK (Fig. 3b). While some specific STs were common in both Oxford and Cairo (ST-21 and ST-50), others were much more common in one specific location, e.g. ST-53 in Oxford, and ST-1519 and ST-3769 in Cairo (Fig. 3c). There was evidence that some lineages associated with AMR (Fig. 3d). While the ST-50 genotype is very common and has been reported more than 3900 times in pubMLST from 40 countries, this is the first report from Africa, to our knowledge. In both Oxford and Cairo datasets, ST-50 was often predicted to be multidrug resistant (MDR). ST-21 is also very common, with more than 4000 reports from 33 countries in pubMLST but was much less likely to be MDR. Four isolates of the Cairo specific ST-3769 also represented a high proportion of MDR (Fig. 3e).

### Extensive multi-drug resistance in local *C. coli* STs

**Fig. 4.**
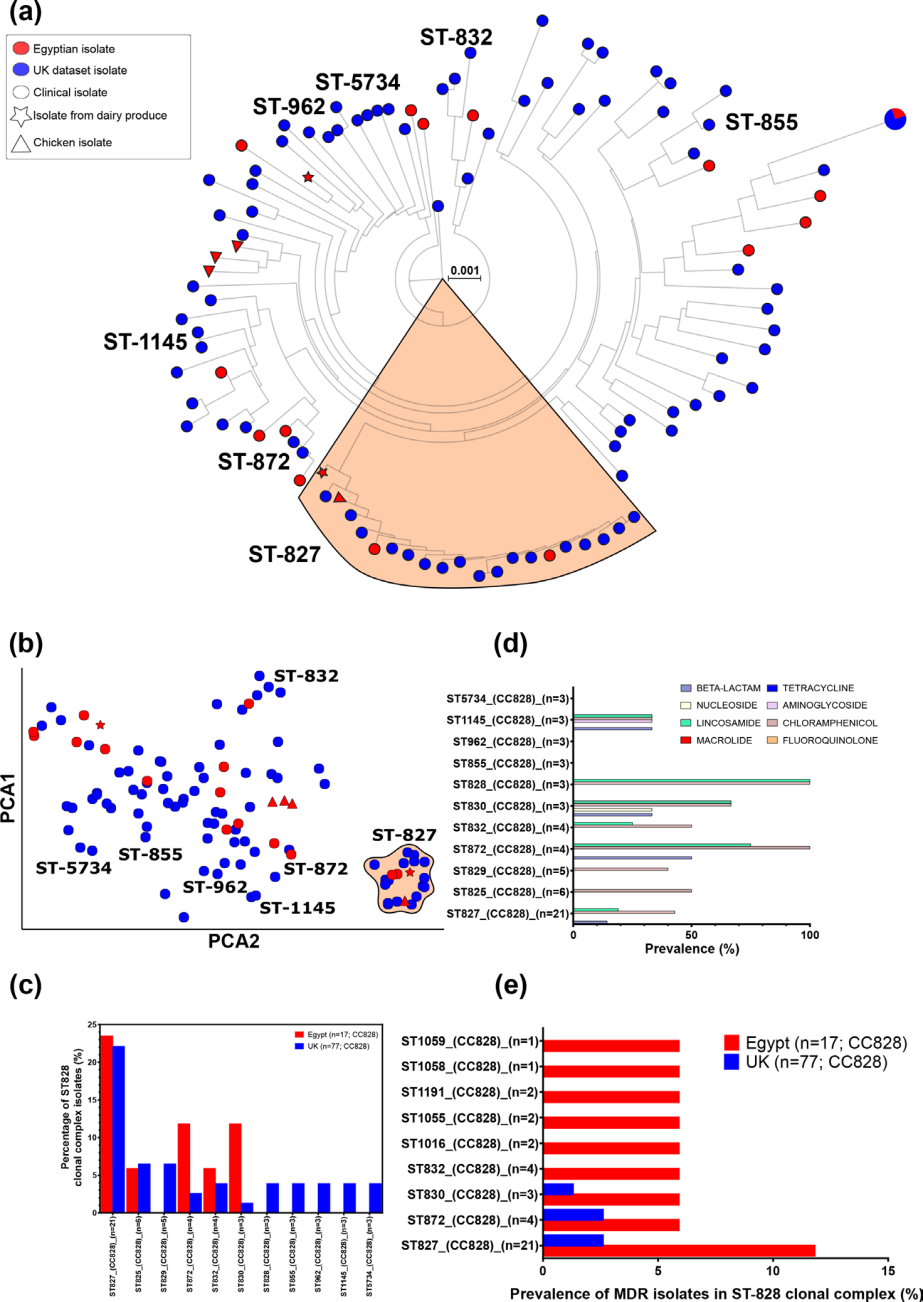
(**a**) Sub-tree of all Egyptian and UK ST-828 CC (CC828) isolates (*n*=94). Common STs are annotated and ST-827 is highlighted (in orange). Scale bar indicated genetic distance. (**b**) Within CC clustering of pairwise core- and accessory-genome distances with PopPUNK according to two principle components. (**c**) Prevalence of the most common STs found within CC828. (**d**) Prevalence of AMR determinants grouped by antibiotic class for each CC828 ST. (**e**) Prevalence of MDR isolates (AMR determinants for three or more antibiotic classes) in CC828 STs.

Greater admixture was noted between UK and Egyptian ST-828 CC isolates than for ST-21 CC – no geographical clustering was observed in either the core or accessory genomes (Fig. 4a, b). However, only ST-827 was common in both datasets (Fig. 4c). Several STs were found in the Oxford dataset that were not identified in our Egyptian isolates, including the frequently isolated STs 829, 828, 855, 962,1145 and 5734. Several lineages were highly resistant to lincosamides, with more than half the isolates from ST-828, ST-830 and ST-872 predicted to be resistant (Fig. 4d). All isolates from ST-828 and ST-872 were also predicted to be resistant to chloramphenicol. Overall, *C. coli* isolates (6 of 105, 5.7%) were far more likely to be considered MDR than *C. jejuni* isolates (6 of 876, 0.68%) and ST-828 complex isolates from Cairo (2 of 19, 10.5%) demonstrated much higher rates of MDR than in Oxford (3 of 77, 3.9 %; Fig. 4e).

### Antimicrobial-resistance genes are distributed across isolates

Each bacterial genome was screened for the presence of genes associated with AMR (Table S3). In Egypt, the mean number of AMR genes per isolate was similar between *C. jejuni* (1.3) and *C. coli* (2.1) (Fig. S2a). The most common AMR determinants identified in Egyptian isolates included the *tet(O*) gene (50 % of all isolates; 61/112), conferring tetracycline resistance, present in 76 and 44 % of *C. coli* and *C. jejuni* isolates, respectively. This pattern contrasts with Oxford where 41 % of *C. jejuni* but only 35 % of *C. coli* isolates were found to harbour *tet(O*) (Table S3). ST-21 CC had a high prevalence of genes associated with β-lactam resistance (particularly the *bla*_OXA-193_, *bla*_OXA-450_ and *bla*_OXA-605_ alleles). *C. coli* held a greater breadth of genes across classes of antimicrobials and the proportion of MDR isolates (resistant to three or more different antibiotic classes) was 28% for *C. coli* compared to 1% for *C. jejuni* [81]. The majority (88%) of MDR isolates in Egypt were *C. coli *, despite *C. coli* representing about a fifth of the dataset (Fig. S2b). The *C. jejuni* isolates that were MDR were all host generalists – ST-21, ST-48 or ST-206. Differences in the presence of AMR genetic determinants of these isolates have been further investigated in another publication, including comparisons with phenotypic susceptibility [43].

## Discussion

Diarrhoeal disease is a major threat to human health and the second leading cause of death in children under 5 years of age in LMICs [8]. Campylobacteriosis is a major cause of diarrhoeal disease worldwide [7, 14, 82] but, despite the potential importance, little is known about *Campylobacter* in countries where it potentially poses the greatest health risk. As studies begin to take a worldview of *Campylobacter* epidemiology and transmission [83], and based on our recent study reporting the AMR phenotypes and genetic determinants among Egyptian *Campylobacter* isolates [43], we aimed in this study to extend our investigation to describe globally disseminated agriculture-associated disease-causing lineages based on core- and accessory-genome content, with evidence that local accessory genome sharing driving acquisition of AMR genes in specific lineages.

The Egyptian *Campylobacter* isolates included a diverse set of STs, including common disease-causing lineages and regional STs that have rarely been reported from other parts of the world. Industrialized agriculture globalization has dispersed livestock worldwide [83], expanding the geographical range of *C. jejuni *. This is evident in the Egyptian collection as two of the most predominant genotypes belonged to the ST-21 and ST-206 CCs (Fig. 1d). These two host generalist CCs have been extensively reported worldwide and frequently isolated from various reservoir hosts, including human clinical samples [22, 29, 55, 76, 84, 85]. The ST-21 CC exhibits considerable genome plasticity with a clear association with several virulence genes and resistance to various antimicrobial agents [86–90]. Poultry-associated CCs, ST-206, ST-464, ST-48, ST-257 and ST-354, were also common among the Egyptian isolates, all of which are among the most prevalent CCs isolated in Europe [91–94].

Further comparison of isolate genotypes collected in Cairo with a large global collection revealed the absence of certain lineages, most notably the lack of the cattle-associated genotype ST-61 [95, 96]. There was only one isolate, of dairy product origin, that could be attributed to a cattle-specialist CC (ST-42), which is unexpected as several (*n*=24) isolates were sampled from dairy products. *Campylobacter* isolates from cattle have predominantly been sampled from meat, milk products and faecal sources (*n*=2726 in pubMLST) [96–98], suggesting that dairy products isolates might represent a different source population in Egypt.

There were also no isolates belonging to the ST-22 CC, a particularly high-risk lineage that is commonly found among patients with post-infectious complications of campylobacteriosis, such as Guillain-Barré syndrome and irritable bowel syndrome [99, 100]. Although one isolate in our collection was from ST-45 CC, this host generalist CC is often one of the most commonly isolates lineages in clinical surveillance studies worldwide [6, 101–103]. Notably, however, it is often absent (or under-represented) in studies conducted in LMICs [71, 104, 105]. This is consistent with observations from other LMICs, where local differences in disease epidemiology are reflected by the absence of common *Campylobacter* lineages, and the presence of rare or unique STs [71, 106–108]. Among our Egyptian isolates, the ST-1287 CC (*n*=2) has been reported less than four times from other parts of the world [91, 101, 108, 109].

Geographical differences have been noted in ST-21 CC [41, 78, 110–113]. ST-21 CC isolates are among the most common *C. jejuni* genotypes isolated worldwide, with one quarter of *C. jejuni* isolates recorded in the pubMLST database are ST-21 CC. Isolates of the ST-50 (*n*=3915) alone have been sampled from 6 continents and 44 countries, although this will be their first report from Africa to our knowledge [56]. Our Egyptian ST-50 isolates do cluster together on a maximum liklihood (ML) phylogeny of ST-21 CC isolates and away from the Oxford ST-21 CC when grouped by PopPUNK. Two STs were unique to Egypt, ST-1519 and ST-3769, with nearly 10 % of the ST-3769 isolates being MDR. A slightly greater proportion of the Egyptian ST-50 isolates were also MDR, although this ST has been observed to be MDR in other parts of the world [114].

The *C. coli* ST-828 CC did not show as much geographical segregation, and when grouping our Egyptian isolates by core- and accessory-genome distances they clustered with the UK clinical isolates, despite several STs being isolated in only one of the datasets. STs found in the Egyptian dataset were more often MDR than UK isolates and, overall, *C. coli* from Cairo were far more MDR than *C. coli* isolates from developed countries [31, 115, 116]. The most compelling clarification for such abundance could be that *C. coli* of ST-828 CC have a great recombination potential besides the accumulation of *C. jejuni* DNA throughout the genome of this lineage, which could have led to the acquisition of multiple AMR genes [117].

Overall, there is clear evidence of local sharing and recent acquisition of accessory-gene content of AMR genes within the Egyptian isolates. Specifically, pairwise clustering of isolates by core and accessory-genome distances recapitulated clusters according to ST and CC (Fig. 2); however, most Egyptian isolates were more tightly clustered than the Oxford clinical dataset, consistent with shared acquisition of accessory genes. Overall, ANI and shared accessory genes were similar between Oxford and Egyptian isolates (per isolate). While temporal variation could influence comparisons, AMR is known to be relatively stable in UK *Campylobacter* isolates [118]. However, the two most common CCs found in our Cairo dataset demonstrated greater sharing of accessory genes, indicative of a shared gene pool. Our study suggested that while geographical partitioning doesn’t impact the composition of the core genome, represented by the shared STs and CCs, the accessory genome is influenced. Within the Egyptian isolates, the most prevalent genotypes (ST-21 CC and ST-206 CC) showed clear evidence of transmission of MDR determinants among lineages. Multiple factors could influence this, such as livestock and food production practices and the segregation of MDR isolates. However, selective pressure for MDR is clearly attributable to antibiotic usage and potentially zoonotic transmissions, as well as the rate of horizontal gene transfer [94]. Our study provides evidence to support programmes aimed at improved antibiotic stewardship in clinical and veterinary settings. With strict control measures, and an understanding of transmission of strains from animal reservoirs through the food production chain, it may be possible to reduce contamination with MDR *Campylobacter* in Egypt.

## Supplementary Data

Supplementary material 1

Supplementary material 2

Supplementary material 3

Supplementary material 4

Supplementary material 5
