# Supplementary material for: Local accessory gene sharing among Egyptian Campylobacter potentially promotes the spread of antimicrobial resistance
Source: Microb Genom. 2022 Jun 8;8(6):mgen000834. doi: 10.1099/mgen.0.000834 (PMC9455717; doi:10.1099/mgen.0.000834)
Supplement: Supplementary material 1 [file mgen-8-834-s001.pdf]

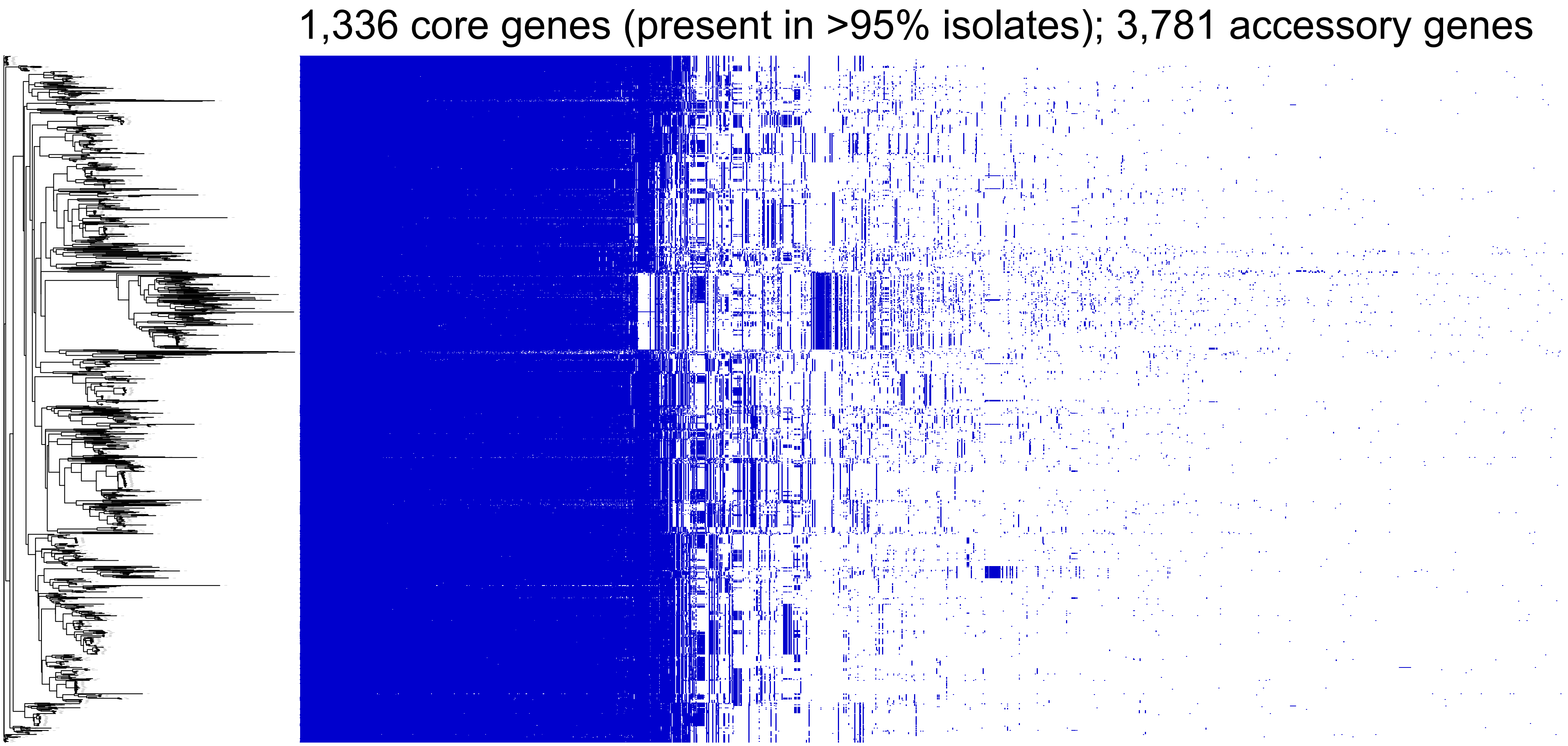

Supplementary figure 1: Visualization of the pangenome (PIRATE) with phandango [50], including estimation of the core (gene present in 95% or more isolates) and accessory genome composition.
