# Supplementary material for: Local accessory gene sharing among Egyptian Campylobacter potentially promotes the spread of antimicrobial resistance
Source: Microb Genom. 2022 Jun 8;8(6):mgen000834. doi: 10.1099/mgen.0.000834 (PMC9455717; doi:10.1099/mgen.0.000834)
Supplement: Supplementary material 2 [file mgen-8-834-s002.pdf]

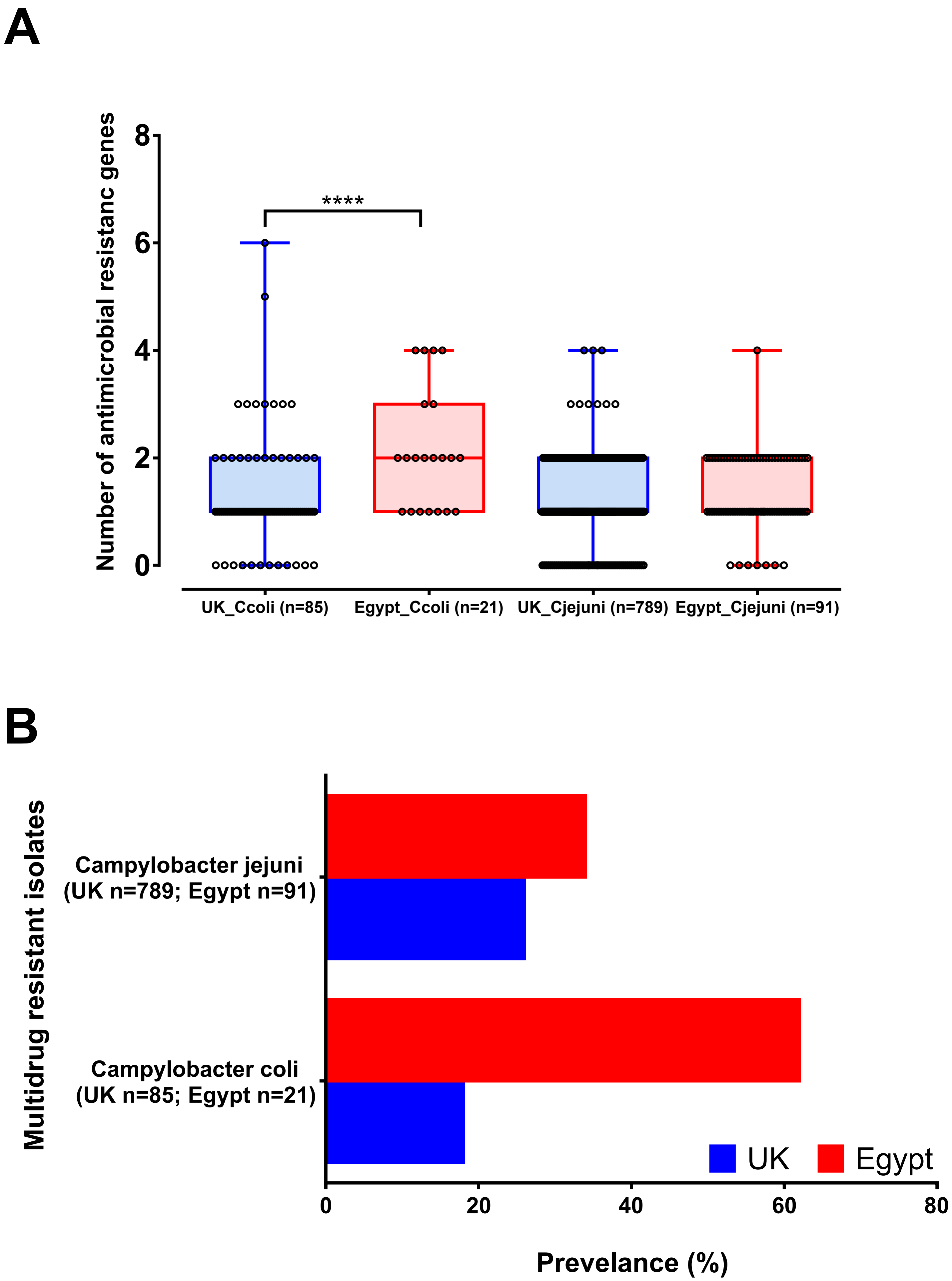

Supplementary figure 2: (**A**) Box plot of the number of antimicrobial resistance genes identified in each *C. jeuni* and *C. coli* population. All isolates shown (circles) and statistical significance indicated by (****); $p<0.0001$; 1-way ANOVA). (**B**) Histogram summarising the prevalence of multidrug resistant isolates in each population.
